# A scoping review of information provided within degenerative cervical myelopathy education resources: Towards enhancing shared decision making

**DOI:** 10.1371/journal.pone.0268220

**Published:** 2022-05-19

**Authors:** Rishi Umeria, Oliver Mowforth, Ben Grodzinski, Zahabiya Karimi, Iwan Sadler, Helen Wood, Irina Sangeorzan, Petrea Fagan, Rory Murphy, Angus McNair, Benjamin Davies

**Affiliations:** 1 Department of Clinical Neurosciences, Division of Neurosurgery, University of Cambridge, Cambridge, United Kingdom; 2 Myelopathy.org, Cambridge, United Kingdom; 3 School of Clinical Medicine, University of Cambridge, Cambridge, United Kingdom; 4 Department of Neurosurgery, Barrow Neurological Institute, Phoenix, Arizona, United States of America; 5 Centre for Surgical Research, Bristol Medical School: Population Health Sciences, University of Bristol, Bristol, United Kingdom; University of Toronto, CANADA

## Abstract

**Background:**

Degenerative cervical myelopathy (DCM) is a chronic neurological condition estimated to affect 1 in 50 adults. Due to its diverse impact, trajectory and management options, patient-centred care and shared decision making are essential. In this scoping review, we aim to explore whether information needs in DCM are currently being met in available DCM educational resources. This forms part of a larger Myelopathy.org project to promote shared decision making in DCM.

**Methods:**

A search was completed encompassing MEDLINE, Embase and grey literature. Resources relevant to DCM were compiled for analysis. Resources were grouped into 5 information types: scientific literature, videos, organisations, health education websites and patient information leaflets. Resources were then further arranged into a hierarchical framework of domains and subdomains, formed through inductive analysis. Frequency statistics were employed to capture relative popularity as a surrogate marker of potential significance.

**Results:**

Of 2674 resources, 150 information resources addressing DCM were identified: 115 scientific literature resources, 28 videos, 5 resources from health organisations and 2 resources from health education websites. Surgical management was the domain with the largest number of resources (66.7%, 100/150). The domain with the second largest number of resources was clinical presentation and natural history (28.7%, 43/150). Most resources (83.3%, 125/150) were designed for professionals. A minority (11.3% 17/150) were written for a lay audience or for a combined audience (3.3%, 5/150).

**Conclusion:**

Educational resources for DCM are largely directed at professionals and focus on surgical management. This is at odds with the needs of stakeholders in a lifelong condition that is often managed without surgery, highlighting an unmet educational need.

## Introduction

In his address to the Royal College of Surgeons in 1923, Rudyard Kipling described words as “the most powerful drug used by mankind”. This metaphor reflects how good communication can have profound effects on clinical decision making [1, 2], the well-being of patients and can improve patient experience of any consultation [3], even if no treatment is offered [4].

Communication is particularly important in chronic conditions such as degenerative cervical myelopathy (DCM) [5, 6], which is estimated to affect 2% of adults [7]. DCM arises when degenerative changes in the cervical spine precipitate a mechanical stress injury of the spinal cord [8]. This leads to progressive disability, including loss of manual dexterity, imbalance, sensory disturbance and pain. Surgery to decompress the spinal cord is the only evidence-based management [9–11]. However, surgery carries risks, may not be required in mild and stable cases, and despite surgery, fewer than 5% of people make a full recovery. Most people with DCM consequently require long-term support for a range of disabilities.

DCM management decisions are therefore complex, individualised [12] and dependent on contextual and technical factors. In particular, the variable and unpredictable trajectory [8], the role of disease surveillance [13] and the importance of managing recovery expectations [14] mandate high-quality communication. Patients must be well informed and have the opportunity to apply and utilise the information that is communicated [15, 16].

Achieving this is challenging. Many factors contribute to effective communication [2], not least selection and prioritisation of information [17], which is prone to bias leading to variability in the quantity or quality of information exchanged [18, 19]. This is pertinent to DCM where complex care pathways involve numerous different professional disciplines [20, 21] often with limited and outdated knowledge of the condition [22, 23]. In addition, the selection of information for resources such as leaflets and websites is commonly driven by the perceptions of healthcare professionals [24].

Providing information effectively for individual consumers requires a person-centred approach, which can be enhanced by the use of communication tools [25]. The objective is to provide the right information, in the right quantity, at the right time, in a manner that can be understood by the individual [26]. This approach is recognised widely in clinical guidelines, including the NHS Person-Centred Approaches Framework [27], the General Medical Council guidance on decision making and consent [28] and is endorsed by the NHS England Personalised Care Institute [29]. Myelopathy.org is specific DCM charity, which was founded to address the challenges faced by the community of people living with DCM [5, 30].

The objective of this scoping review was to undertake a structured exploration of the information provided by current DCM educational resources. The aim is to facilitate development of additional tools to support communication in DCM, forming part of a larger Myelopathy.org initiative to promote shared decision making in DCM.

## Methods

### Resource type categorisation

DCM educational resources were categorised into 5 types: scientific literature, videos, health organisations, health education websites and hospital information leaflets. Consensus on resource types was reached by the authorship group, which included people with DCM. Health seeking behaviour of the public and health information provision from healthcare professionals was considered when developing the categorisation system [31]. For example, healthcare professionals frequently distribute patient information leaflets (PILs) [32, 33] and the public increasingly access health education and health organisation websites and online videos [34–36].

### Search strategy

A comprehensive search strategy was developed and refined for each of 7 key resource domains (S1 Appendix). For the health organisation and health education websites, a hierarchical search strategy was employed to identify DCM educational content (Table 1).

**Table 1:** Search strategy for organisations, health education websites and hospital patient information leaflets.

Method	Tool	Search term	Additional information
1	Select webpage on patient or professional resources from navigation menu	Cervical myelopathy	N/A
2	Searchbar on website	Cervical myelopathy	Search performed once for a given website
3	Find in page function (Ctrl + F)	Cervical myelopathy	Search repeated for each webpage page on a given website

Hierarchical search strategy to identify educational content on DCM from health organisation websites, health education websites and hospital patient information leaflets. Method 1 was employed first. Method 2 was employed if no information on DCM was found using method 1. Method 3 was used employed if no information was found using methods 1 and 2. If no information found using all 3 methods, the resources was excluded.

Overall inclusion and exclusion criteria were created (Table 2) and adapted for each specific resource type (S2 Appendix). Educational content exclusively addressing degenerative cervical myelopathy was sought. Content covering cervical myelopathy of non-degenerative aetiology was excluded.

**Table 2:** Overall criteria for screening for DCM educational resources.

Inclusion Criteria	Exclusion Criteria
English language	Heterogenous populations (not exclusively DCM or CSM +/- OPLL)
Educational tool	Cervical myelopathy of non-degenerative aetiology
Degenerative cervical myelopathy OR Cervical spondylotic myelopathy +/- OPLL	Cervical radiculopathy

Overall inclusion and exclusion criteria for screening resources to identify those with educational DCM content. These criteria were applied to screen resources of from all 5 key resource types: scientific literature, videos, health organisations, health education website and hospital patient information leaflets. The aim was to identify public-facing resources that contained educational content on DCM. Specific inclusion and exclusion criteria were then adapted for each resource type (S2 Appendix).

### Resource types

#### 1. Scientific literature

High sensitivity DCM search filters were used to search the databases EMBASE [37] and MEDLINE [38] (S3 Appendix). The PROSPERO database, a prospectively maintained register of planned systematic reviews, was also searched using the term “myelopathy”. Searches were performed on 6^th^ May 2020. To identify currently relevant information, searches were limited to the past 3 years for narrative reviews and the past 20 years for systematic reviews. These article types were selected because narrative reviews typically aim to provide educational content and systematic reviews focus on specific clinical questions.

#### 2. Videos

Google is the most popular search engine, accounting for 93% of all internet searches [39]. A search of ‘cervical myelopathy’ was run under the *videos* subsection of Google on 23^rd^ April 2020 to identify educational videos on DCM. In total, 54% of videos (15/28) returned in the search were from YouTube and 46% (13/28) of videos were from other multimedia websites.

#### 3. Organisations

A comprehensive global list of organisations with potentially relevant educational content on DCM were identified by the AO Spine RECODE-DCM ENVIROSCAN project. This project is collaborative research effort, aiming to increase DCM research efficiency [40]. The ENVIROSCAN is diverse list of organisations–charities, hospitals, universities and professional academic bodies–either involved in, or with the potential to be involved in, DCM research. A hierarchical search strategy was used to search 737 organisation websites (Table 1).

#### 4. Health education websites

Alexa Top Sites, part of Amazon Web Services, was used to identify the most popular websites under the category of health, subcategory education, based on website traffic on 6^th^ May 2020 [41]. The top sites feature of Alexa uses a traffic rank algorithm based on relative reach and page views over the previous three months by Alexa users to determine the highest reaching websites. The hierarchical search strategy was used to search the top 50 health education websites.

#### 5. Hospital patient information leaflets

A list of the hospitals recorded as specialised providers of complex spinal surgery from Appendix C of the spinal services report (S4 Appendix) [42], captures all hospitals in the United Kingdom offering surgical treatment for DCM [11, 20]. Our hierarchical search strategy was used to search 40 hospital websites for information on DCM provided by the hospital.

### Data extraction

Educational information was extracted from included resources by one author (RU) at two separate time points, to ensure all educational content was extracted.

### Resource domain development

Extracted information was then categorised inductively by two authors (RU and BMD) into 7 key information domains: aetiology, pathophysiology and epidemiology; clinical presentation and natural history; diagnosis and monitoring progression; surgical management; non-surgical management; predicting outcomes; assessing research and developing guidelines (Table 3). Domains were developed independently by assessing all information resources and iteratively refined until they were applicable across all content, comprehensively covered all key concepts and consensus between authors was reached. One author (BMD) had prior knowledge about DCM, whilst the other author (RU) did not, meaning the final categorisation system reflected a reconciliation between a knowledge-driven and an unbiased approach. Agreed information domains were further divided into a framework of information subdomains to enable content analysis of information from different resources using descriptive statistics (Table 4). Additional criteria were used to determine if each resource was targeted at a lay or professional audience or both (Table 5). A specific distinction of whether a resource was for patients or other lay stakeholders was not attempted.

**Table 3:** Domain categorisation system for DCM educational resources.

Domain number	Domain name
1	Aetiology, pathophysiology and epidemiology
2	Clinical presentation and natural history
3	Diagnosis and monitoring progression
4	Surgical management
5	Non-surgical management
6	Predicting outcomes
7	Assessing research and developing guidelines

Seven key domains were identified to categorise educational content on DCM in to.

**Table 4:** Summary of DCM educational resources categorisation.

Title	Code	Narrative Reviews (16)		Systematic Reviews (99)		Videos (28)		Organisations (5)		Health Education Websites (2)	
Aetiology, pathophysiology and epidemiology	1	12	75%	6	6%	11	39%	2	40%	2	100%
Aetiology	1a	2	13%	4	4%	1	4%	0	0%	0	0%
Pathophysiology	1b	11	69%	2	2%	10	36%	2	40%	2	100%
Epidemiology	1c	11	69%	0	0%	0	0%	0	0%	0	0%
Clinical presentation and natural history	2	11	69%	6	6%	20	71%	4	80%	2	100%
Symptoms	2a	10	63%	0	0%	18	64%	4	80%	2	100%
Signs	2b	8	50%	1	1%	7	25%	0	0%	0	0%
Natural history	2c	10	63%	5	5%	12	43%	4	80%	2	100%
Diagnosis and monitoring progression	3	12	75%	11	11%	7	25%	3	60%	1	50%
Diagnosis	3ai	10	63%	8	8%	7	25%	3	60%	1	50%
Monitoring progression	3aii	4	25%	2	2%	0	0%	0	0%	0	0%
Clinical assessment	3bi	10	63%	6	6%	7	25%	3	60%	1	50%
Radiological assessment	3bii	10	63%	1	1%	5	18%	0	0%	0	0%
Electrophysiological assessment	3biii	7	44%	1	1%	1	4%	1	20%	0	0%
Biomarker assessment	3biv	3	19%	1	1%	0	0%	0	0%	0	0%
Surgical management	4	11	69%	61	62%	22	79%	4	80%	2	100%
Surgical approach decision	4a	8	50%	0	0%	6	21%	0	0%	0	0%
Surgical procedure—anterior only	4bi	0	0%	14	14%	1	4%	0	0%	0	0%
Surgical procedure—posterior only	4bii	0	0%	19	19%	1	4%	0	0%	0	0%
Surgical procedure—both	4biii	8	50%	25	25%	9	32%	1	20%	1	50%
Surgical procedure—neither	4biv	3	19%	0	0%	11	39%	3	60%	1	50%
Surgical outcomes	4c	10	63%	61	62%	6	21%	1	20%	0	0%
Non-surgical management	5	6	38%	4	4%	3	11%	2	40%	1	50%
Physiotherapy	5ai	3	19%	4	4%	2	7%	1	20%	1	50%
Medications	5aii	6	38%	2	2%	1	4%	1	20%	1	50%
Cervical traction	5aiii	5	31%	3	3%	0	0%	1	20%	0	0%
Cervical bracing	5aiv	5	31%	1	1%	1	4%	1	20%	1	50%
Bedrest	5av	3	19%	0	0%	0	0%	0	0%	0	0%
Avoidance of risky activities/environment	5avi	2	13%	0	0%	0	0%	0	0%	0	0%
Orthoses	5avii	1	6%	2	2%	0	0%	0	0%	0	0%
No specific interventions (unspecified)	5aviii	1	6%	1	1%	1	4%	0	0%	0	0%
Non-surgical outcomes	5b	5	31%	4	4%	0	0%	0	0%	0	0%
Predicting outcomes	6	7	44%	19	19%	1	4%	0	0%	0	0%
Clinical predictors	6ai	6	38%	9	9%	1	4%	0	0%	0	0%
Imaging predictors	6aii	7	44%	10	10%	0	0%	0	0%	0	0%
Non-specified predictors	6aiii	0	0%	2	2%	0	0%	0	0%	0	0%
Surgical outcomes	6bi	6	38%	15	15%	1	4%	0	0%	0	0%
Non-surgical outcomes	6bii	1	6%	1	1%	0	0%	0	0%	0	0%
Natural disease course Outcomes	6biii	1	6%	9	9%	0	0%	0	0%	0	0%
Assessing research and developing guidelines	7	3	19%	5	5%	1	4%	1	20%	0	0%
Future directions	7ai	3	19%	0	0%	0	0%	0	0%	0	0%
Reporting outcome measures	7aii	0	0%	3	3%	0	0%	0	0%	0	0%
Reporting trends	7aiii	0	0%	1	1%	0	0%	0	0%	0	0%
Developing guidelines	7b	0	0%	1	1%	1	4%	1	20%	0	0%

For each resource type the number of resources in the domain and subdomain is recorded and the percentage of resources containing information on the domain or subdomain, of all the resources of that type. A single resource can contain information on more than one domain or subdomain within a domain.

**Table 5:** Criteria for target audience determination for DCM educational resources.

Patient Criteria	Professional Criteria
Information delivered in style for individual from non-healthcare background, including use of simple language (avoiding medical jargon)	Information delivered in style for individual from healthcare background, including use of medical jargon, clinical management applications, research study applications
Pre-determined audience by source nature e.g. patient information leaflet (PIL)	Pre-determined audience by source nature e.g. scientific literature
Source content providing information for an individual with DCM on symptoms to look out for, what to expect through interactions with healthcare professionals for accessing treatment pathway, how they can adapt to living with their condition	Information delivered to audience with a presumed knowledge of anatomy, physiology or pathology of the spine and spinal cord
	Source content providing information on how to take a history, perform a physical examination, investigations to order or approach to management of an individual with DCM

Criteria to determine the target audience of each resource: patients, professionals or a combined audience Resources that met components of both the patient and professional criteria were recorded as having a combined audience.

## Results

**Fig 1:**
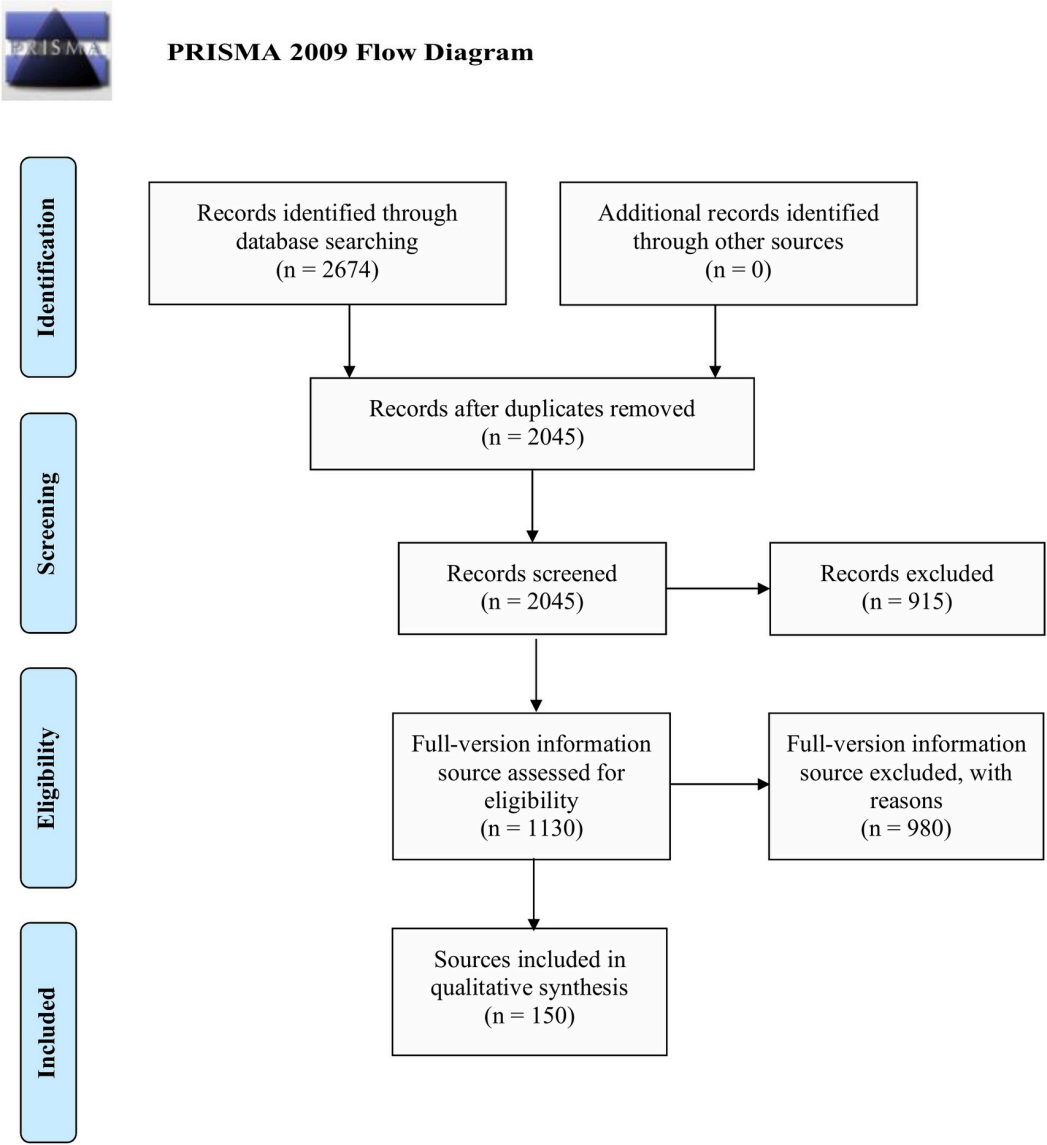
PRISMA (Preferred Reporting Items for Systematic Reviews and Metanalyses) flow diagram of search strategy. The process of identifying the educational resources that met inclusion criteria from databases illustrated as a flow diagram.

Of the 2674 resources screened, 150 DCM information resources were identified: 115 from the scientific literature, 28 videos, 5 from organisations and 2 from health education websites (Fig 1). No hospital information leaflets were identified. Reporting for this review followed the Preferred Reporting Items for Systematic Reviews and Meta-analyses (PRISMA) checklist (S5 Appendix).

**Fig 2:**
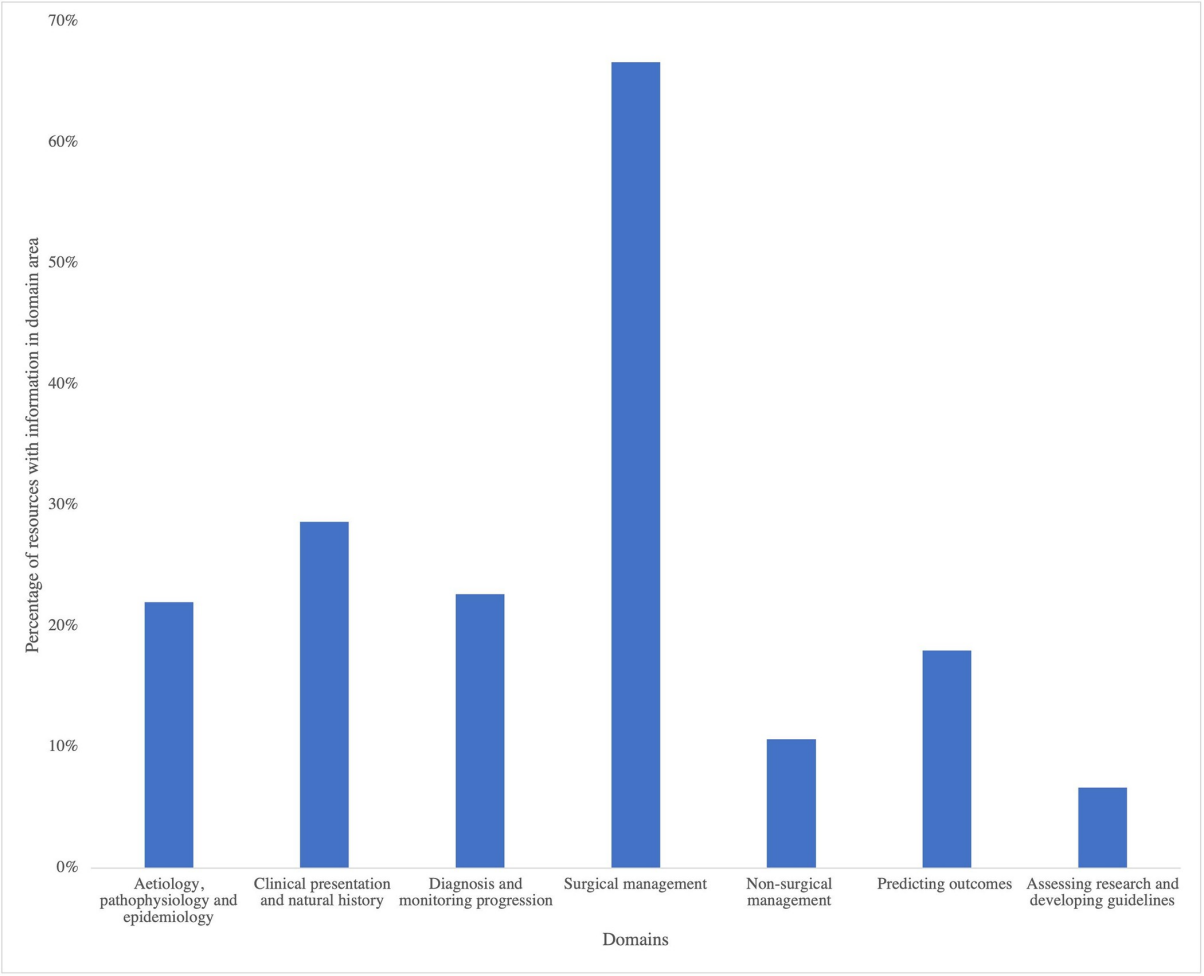
Percentage of educational resources on each of the 7 DCM domains. The most common domain was surgical management (67%). The least common domain was assessing research and developing guidelines (7%).

The most common domain addressed was surgical management, with 67% (100/150) of resources. The least common domain was assessing research and developing guidelines, with 7% (10/150) of resources (Fig 2). Approximately 11% (16/150) of resources covered nonsurgical management. The majority of resources (86%, 129/150) were designed for a professional audience; a minority were targeted at a lay audience (11%, 16/150) or a joint professional and lay audience (3%, 5/150).

### Domain 1—Aetiology, pathophysiology and epidemiology (33 resources)

Key themes identified were that the disease process is poorly understood, however there appears to be consensus that both static and dynamic injury contribute to chronic cervical cord compression. Most resources in this domain (82%, 27/33) discussed DCM pathophysiology, including ischaemia, disruption of the spinal cord-blood barrier, inflammation and apoptosis.

A total of 12% (4/33) of resources in this domain consider the role of genetic factors in DCM, such as the fact that only small percentage of individuals with radiological evidence of cervical cord compression have clinical features of DCM, may represent a genetic susceptibility to DCM.

In total, 33% (11/33) of resources addressed epidemiology. Scientific literature resources in particular, often report DCM as the leading cause of spinal cord dysfunction in adults worldwide, however, frequently lack precise estimates of prevalence and incidence. Common reasons cited include the lack of a standardised definition of DCM, difficulty in diagnosis, and a lack of large-scale observational studies. Myelopathy.org reports that up to 5% of people over the age of 40 may have DCM [43]. Age is widely cited as an important risk factor, with degenerative changes being more common with age and most people with DCM being diagnosed in their 50s. An important consideration is how the incidence of DCM may increase with a globally ageing population [44].

### Domain 2—Clinical presentation and natural history (43 resources)

A total of 79% (34/43) of resources in this domain described the symptoms of DCM, with fewer reporting on signs (37%, 16/43). Clinical presentation was often covered thoroughly describing the classical presentation of neck pain or stiffness, poor manual dexterity, clumsiness, paraesthesia, gait dysfunction and bladder and bowel dysfunction. Over 75% (33/43) of resources in this domain reported on the natural history of DCM, often commenting on the variability and unpredictability. A frequent statistic cited by several resources, is that 20% to 62% of people with DCM experience clinical deterioration (defined by a change of at least one point in the modified Japanese Orthopaedic Association (mJOA) score over a 3-to-6-year follow-up period [45].

### Domain 3—Diagnosis and monitoring progression (34 resources)

Diagnosis was discussed by 85% (29/34) of resources in this domain. A key theme addressed was the need for specialist involvement, including the importance for doctors in primary healthcare to refer a person with suspected DCM to a spinal surgeon, who may fall under the remit of neurosurgery or orthopaedics, for a specialist assessment.

Radiological assessment and clinical assessment were discussed by 47% (16/34) and 79% (27/34) of resources respectively. The combination of MRI of the cervical spine alongside clinical signs and symptoms was an important point frequently made regarding diagnosis. There is also consensus that DCM is difficult to diagnose and that there is poor awareness of the condition among the general public and non-specialist healthcare professionals.

The topic of monitoring progression, reported on by 18% (6/34) resources, involved commentary that different resources refer to different tools to assess functional impairment in DCM. This has led to inconsistencies in assessing outcomes. The mJOA scale and Nurick scale are commonly used DCM-specific indices, yet both have poor sensitivity [46–48].

### Domain 4—Surgical management (100 resources)

This domain was covered by 67% (100/150) of all resources. Key themes were that surgery aims primarily to decompress the spinal cord and aims secondarily to stabilise the spinal column. Common anterior and posterior surgical approaches, such as anterior cervical discectomy and fusion and laminectomy were frequently discussed. A total of 15% (15/100) of resources in this domain reported on anterior approach only, 20% (20/100) on posterior only, 44% (44/100) on both anterior and posterior, and 18% (18/100) discussed surgical procedure more generally. A key theme covered by 14% (14/100) of resources, including both videos and scientific literature, were factors that determine surgical approach. Systematic reviews were the most common resource type (61%, 61/100) to report on surgical management.

Surgical outcomes, including efficacy and complications of surgery, were discussed by 78% (78/100) of resources in this domain. Another frequently discussed theme was the role of surgery in stopping the progression of the disease and preventing further neurological decline. A small number of scientific literature resources cited recently growing evidence that surgery may improve neurological function. Resources frequently refer to clinical guidelines published by AO Spine in 2017, which strongly recommend surgery for moderate to severe DCM and recommend a structured trial of rehabilitation or surgery for mild DCM [11]. Surgical complications were another key theme. For the anterior approach this included dysphagia, damage to local structures, pseudoarthrosis and for the posterior approach it included C5 palsy and surgical site infection.

### Domain 5—Non-surgical management (16 resources)

There was considerably lower coverage of the nonsurgical management domain compared to the surgical management domain. A total of 63% (10/16) of nonsurgical management resources were from the scientific literature. Resources described the consensus that nonoperative management does not provide definitive treatment for spinal cord compression but may have a role in symptomatic management for mild DCM. Nonoperative management discussed included physiotherapy (69%, 11/16), medication (69%, 11/16), cervical traction (56%, 9/16), orthoses (19%, 3/16), bedrest (19%, 3/16) and avoidance of high-risk activities (13%, 2/16). In total, 56% (9/16) of resources in this domain discussed outcomes of non-surgical management, all of which were from the scientific literature. The lack of high-quality evidence to support the role of non-surgical management was commonly discussed.

### Domain 6—Predicting outcomes (27 resources)

Predicting outcomes was covered by 18% (27/150) of resources. All resources were from the scientific literature, except for one video, discussing modelling outcome prediction in DCM. A key theme was the use of clinical data and imaging data to predict outcomes, discussed by 59% (16/27) and 63% (17/27) of resources, respectively. Literature resources discussed factors that may determine if a person is likely to benefit from surgery; surgical outcomes were discussed by 81% (22/27) of resources in this domain, while outcomes from non-operative management were discussed by 7% (2/27) of resources. Commonly cited factors associated with poorer surgical outcomes include older age, longer symptom duration, and worse preoperative disease severity [49]. Predicting outcomes in the natural disease course was addressed by 37% (10/27) of resources in this domain. This appears to be an important theme for people with DCM, as predicting outcomes can manage a person’s expectations [14] and lead to expeditious referral for people with predictors of rapid deterioration.

### Domain 7—Assessing research and developing guidelines (10 resources)

In total, 7% (10/150) of resources provided information on assessing research and developing guidelines. An important theme was inconsistency in terminology and outcomes reducing the efficiency of research, which was covered by 30% (3/10) of resources in this domain. Future research to further the field was discussed by 30% (3/10) of resources. Advances in imaging, including quantitative MRI, and the use of biomarkers are two key upcoming areas. Another theme is addressing knowledge gaps about DCM, for example with respect to clinical practice guidelines the optimal treatment strategy for mild DCM remains unknown [11].

## Discussion

Following a comprehensive search, 150 DCM educational resources were analysed. The majority were targeted at professionals, rather than a lay audience. Information provision largely focused on surgical management and to a lesser extent clinical presentation.

### Dominance of the scientific literature and surgery

More than three-quarters (77%, 115/150) of all resources identified came from the scientific literature, this included systematic reviews (66%, 99/150) and narrative reviews (11%, 16/150). The two domains with the highest proportion of resources coming from the scientific literature were domain 6 on predicting outcomes (96%, 26/27) and domain 7 on assessing research and developing guidelines (80%, 8/10). Of all 115 resources from the scientific literature, the two most common domains covered were domain 4 on surgical management (63%, 72/115) and domain 6 (23%, 26/115). As educational content from the scientific literature is directed at healthcare professionals, it is not surprising that there is a focus on surgery, the main treatment modality for DCM. The content is especially important for informing guidelines and making evidence-based decisions on the surgical approach for spinal decompression [50].

Of all 72 resources from the scientific literature that covered surgical management, 71 (99%) addressed outcomes of surgery, compared to 27% (6/22) of videos, 25% (1/4) of resources from organisation and 0% (0/2) of resources from health education websites. Only 4% (3/72) of literature resources on surgical management discussed surgical procedures in general, non-technical terms, whereas non-literature resources were more likely to: 50% (11/22) of videos, 75% (3/4) of organisations, 50% (1/2) health education websites discussed surgery more generally.

For the 28 video resources, surgical management was the most common domain (79%, 22/28) and the second most common domain was clinical presentation and natural history (71%, 20/28). Videos were targeted at the general public and professionals with equal frequency. Understanding how to recognise the disease and explaining how it is managed were common notable features of videos. The small number of organisations and health education websites identified, limited depth analysis of these resource types and highlights the paucity of DCM educational resources.

### Lack of information on DCM

The total of 150 educational resources identified within the parameters of this review, highlights the limited educational resources available on DCM, especially for a lay audience (11%, 16/150). Possible factors that may be contributing include the variable terminology used for the condition and the fact that there are many aspects of DCM that are not well understood. These factors may create barriers for development of educational resources since answers to certain questions about the condition simply do not exist. The AO Spine RECODE-DCM project has established global research priorities to address knowledge gaps in the field and this will help coordinate the research effort to tackle this issue [40].

All domains, except surgical management, were covered by less than 30% of the educational resources. Domain 1 coverage was particularly low (22%, 33/150), which may be due to poor understanding of DCM aetiological factors, pathophysiological mechanisms, and lack of large epidemiological datasets to inform accurate estimates of incidence and prevalence. Furthermore, domain 2 coverage may be low (29%, 43/150) due to the variability in initial presentation and disease progression. Moreover, low domain 3 coverage (23%, 34/150) may be because DCM is difficult to diagnose, especially early in the disease course, where symptoms may be non-specific, making appropriate educational resources challenging to produce.

Explaining monitoring of progression in resources can be a challenge when there is a lack of consensus on what tools should be used. Very poor coverage of domain 5 (11%, 16/150), is likely due to the lack of evidence supporting non-surgical management in DCM, making it difficult to justify the inclusion in educational resources, especially when focusing on evidence-based management. Domain 6 coverage being low (18%, 27/150), might again be explained by the paucity of evidence for outcome prediction, in particular identifying factors that will determine which patients will be most likely to benefit from surgery. Domain 7 (7%, 10/150) had the lowest coverage. This is partly due to this being almost exclusively the remit of scientific literature. Assessing patterns and commenting on the progress of research in the field is challenging when the field of DCM research is still relatively small [51].

Resources from outside the scientific literature were in the minority (23%, 35/150). This aligns with the paucity of resources targeted at a lay audience, who are generally less likely to obtain their information on DCM from the scientific literature [31, 52]. As the DCM research field grows, there is potential for information from the scientific literature to be filtered into other types of resources that are more likely to reach the lay audience.

### Generalisability

A focus on surgery is consistent with the wider scientific literature [53]. This is understandable given that surgery is currently the only disease modifying therapy [54]. However experience from AO Spine RECODE DCM [40] and related projects has demonstrated that this will overlook critical perspectives [55]. For example, a survey by Myelopathy.org identified that pain was the number one recovery priority for people living with DCM [56], yet research to date has rarely measured it [57, 58]. Furthermore, a focus on surgery does not address the large proportion of people with DCM who are currently managed non-operatively [59, 60], the many different professionals involved in this process, [20] and the long-term implications that persist following surgery [61, 62].

This review therefore indicates that education gaps exist within DCM, due to its paucity but also lack of breadth. However, what this review does not characterise is the information need of people living with DCM. This is the objective of a qualitative study being undertaken in parallel by Myelopathy.org.

With this information, the aim is to develop solutions to support personalised information exchange in DCM. One approach may be the formation of core information sets (CIS)—refined lists of critical topics to be discussed, for example in supporting informed consent for surgery [25]. CIS are formed using a multi-stakeholder consensus process, which involves initially gathering information using literature reviews and interviews, before distilling this into a core list of information. CIS are therefore designed to be a starting point to help professionals, patients and their carers ensure key information is considered. They are intended to be personalised during each consultation and have been pioneered in surgical oncology [63–65].

CIS likely have value far beyond surgical consent. In DCM, patients often share common or stereotyped transitions points in their care, such as obtaining diagnosis or preparing for surgical treatment. This makes a series of CIS a potentially effective adjuvant to improve communication and outcomes in DCM. The information domains identified in this review may align well with this. Furthermore, Myelopathy.org has had success using this methodological approach in AO Spine RECODE DCM [20, 40, 65–67]. The definitive next steps will need to be considered in the context of the information needs identified amongst people living with DCM.

### Limitations

Resources were selected pragmatically using a multi-stakeholder perspective and searches were only conducted online. Relevant educational resources, including physical content such as printed information leaflets and local resources not freely available online may therefore not have been captured. The consequence of this, on the conclusions drawn in the article, is unknown. Patients are now known to use the internet as their predominant source of healthcare information over healthcare professionals [68, 69]. Nonetheless, it is difficult to say with certainty the impact printed PILs have on patient behaviour as it depends on the context of the clinical situation and the invasiveness of the intervention [32, 70].

The literature search was performed in May 2020, identifying pre-COVID-19 pandemic data on educational resources. Resources produced during the pandemic will not have been captured. However, the pre-pandemic resources are more likely to be consistent with addressing the research question and help orienting clinical research and the formation of CIS once the COVID-19 pandemic is over.

Furthermore, our inclusion criteria stipulated information sources should be specifically focused on DCM. DCM information may have been grouped with other medical conditions such as cervical radiculopathy, as well as being placed in the broader category of non-traumatic spinal cord injury [71, 72]. This appears a particular issue with hospital patient information leaflets; 10 leaflets providing generic information for cervical surgery were identified as surgical procedures used to treat DCM are common to many other diseases.

However, this is unlikely to be a limitation for DCM CIS. Firstly, owing to a poor awareness of DCM amongst the general public and the general medical community [73, 74], the identification of relatively few printed educational resources was expected. This knowledge gap led to the foundation of Myelopathy.org, which receives an international audience. Moreover, the search covered all resources recognised as relevant to patients seeking health information [31, 75]. Finally, this review will be supplemented by qualitative studies, such as interviews, to identify relevant information for a CIS. This is likely to be of greater and specific relevance to produce patient centred CIS [25].

## Conclusion

There are few dedicated educational resources for people with DCM or the general public. The majority of education material is found within the scientific literature for a professional audience. Key areas of focus included surgical management; clinical presentation and natural history; non-surgical management; and diagnosis and monitoring for progression. Aetiology, pathophysiology, epidemiology, predicting outcomes and developing guidelines were also addressed by professionally orientated resources. This information will be used to inform a larger initiative by Myelopathy.org to support patient centred care in DCM.

## Supporting information

S1 Appendix Search strategy identifying educational resources in scientific literature, videos, organisations, health education websites and hospital patient information leaflets. (DOCX)

S2 Appendix Inclusion and exclusion criteria for educational resources in scientific literature, videos, organisations, health education websites and hospital patient information leaflets. (DOCX)

S3 Appendix DCM search filters for scientific literature databases: EMBASE and MEDLINE. (DOCX)

S4 Appendix List of UK hospitals offering complex spinal surgery services–taken from Spinal Services GIRFT Programme National Specialty Report Appendix C. (DOCX)

S5 Appendix PRISMA checklist. (DOC)

## References

[1] Barrett S, Komaromy C, Robb M, Rogers A. Communication, Relationships and Care: A Reader [Internet]. Google Books. Routledge; 2004. Available from: https://books.google.co.uk/books?hl=en&lr=&id=3Ph-AgAAQBAJ&oi=fnd&pg=PA55&dq=historical+origins+of+counselling&ots=C4EGWJo1V4&sig=gDL08CBRZZ82fI1plKYe2Rx6Bhw#v=onepage&q=historical%20origins%20of%20counselling&f=false

[2] Elwyn G, Frosch D, Thomson R, Joseph-Williams N, Lloyd A, Kinnersley P, et al. Shared Decision Making: A Model for Clinical Practice. Journal of General Internal Medicine [Internet]. 2012 May 23 [cited 2019 Mar 18];27(10):1361–7. Available from: https://www.ncbi.nlm.nih.gov/pmc/articles/PMC3445676/ doi: 10.1007/s11606-012-2077-6 22618581 PMC3445676

[3] Carr AJ. Measuring quality of life: Is quality of life determined by expectations or experience? BMJ. 2001 May 19;322(7296):1240–3. doi: 10.1136/bmj.322.7296.1240 11358783 PMC1120338

[4] Donovan JL. Qualitative study of interpretation of reassurance among patients attending rheumatology clinics: “just a touch of arthritis, doctor?” BMJ. 2000 Feb 26;320(7234):541–4. doi: 10.1136/bmj.320.7234.541 10688559 PMC27296

[5] Davies B, Sadler I, Umeria R. Education & Myelopathy—Knowledge is power? [Internet]. 2021. Available from: https://podcasts.apple.com/gb/podcast/s2-ep5-education-myelopathy-knowledge-is-power/id1493647316?i=1000525442039

[6] Davies B, Kotter M, Fehlings M, Milligan J, Blizzard T. AO Spine Research Top 10—No. 1—Raising Awareness [Internet]. Myelopathy Matters; 2020. Available from: https://podcasts.apple.com/gb/podcast/ao-spine-research-top-10-no-1-raising-awareness/id1493647316?i=1000495367740

[7] Smith SS, Stewart ME, Davies BM, Kotter MRN. The Prevalence of Asymptomatic and Symptomatic Spinal Cord Compression on Magnetic Resonance Imaging: A Systematic Review and Meta-analysis. Global Spine Journal. 2020 Jun 24;219256822093449. doi: 10.1177/2192568220934496 32677521 PMC8119927

[8] Davies BM, Mowforth OD, Smith EK, Kotter MR. Degenerative cervical myelopathy. BMJ. 2018 Feb 22;k186. doi: 10.1136/bmj.k186 29472200 PMC6074604

[9] Fehlings MG, Wilson JR, Kopjar B, Yoon ST, Arnold PM, Massicotte EM, et al. Efficacy and Safety of Surgical Decompression in Patients with Cervical Spondylotic Myelopathy. The Journal of Bone & Joint Surgery. 2013 Sep;95(18):1651–8. 24048552 10.2106/JBJS.L.00589

[10] Fehlings MG, Ibrahim A, Tetreault L, Albanese V, Alvarado M, Arnold P, et al. A Global Perspective on the Outcomes of Surgical Decompression in Patients With Cervical Spondylotic Myelopathy. Spine. 2015 Sep;40(17):1322–8. doi: 10.1097/BRS.0000000000000988 26020847

[11] Fehlings MG, Tetreault LA, Riew KD, Middleton JW, Aarabi B, Arnold PM, et al. A Clinical Practice Guideline for the Management of Patients With Degenerative Cervical Myelopathy: Recommendations for Patients With Mild, Moderate, and Severe Disease and Nonmyelopathic Patients With Evidence of Cord Compression. Global Spine Journal. 2017 Sep;7(3_suppl):70S83S. 10.1177/2192568217701914 PMC5684840 29164035

[12] Khan O, Badhiwala JH, Grasso G, Fehlings MG. Use of Machine Learning and Artificial Intelligence to Drive Personalized Medicine Approaches for Spine Care. World Neurosurgery. 2020 Aug;140:512–8. doi: 10.1016/j.wneu.2020.04.022 32797983

[13] Pope DH, Mowforth OD, Davies BM, Kotter MRN. Diagnostic Delays Lead to Greater Disability in Degenerative Cervical Myelopathy and Represent a Health Inequality. Spine. 2020 Mar 15;45(6):368–77. doi: 10.1097/BRS.0000000000003305 31658234

[14] Wilson JRF, Badhiwala JH, Jiang F, Wilson JR, Kopjar B, Vaccaro AR, et al. The Impact of Older Age on Functional Recovery and Quality of Life Outcomes after Surgical Decompression for Degenerative Cervical Myelopathy: Results from an Ambispective, Propensity-Matched Analysis from the CSM-NA and CSM-I International, Multi-Center Studies. Journal of Clinical Medicine. 2019 Oct 17;8(10):1708. doi: 10.3390/jcm8101708 31627303 PMC6833063

[15] Michie S, Atkins L, West R. The behaviour change wheel: a guide to designing interventions. London: Silverback Publishing; 2014.

[16] Rees S, Williams A. Promoting and supporting self-management for adults living in the community with physical chronic illness: A systematic review of the effectiveness and meaningfulness of the patient-practitioner encounter. JBI Database of Systematic Reviews and Implementation Reports. 2009;7(13):492–582. doi: 10.11124/01938924-200907130-00001 27819974

[17] Bomhof-Roordink H, Gärtner FR, Stiggelbout AM, Pieterse AH. Key components of shared decision making models: a systematic review. BMJ Open [Internet]. 2019 Dec;9(12):e031763. Available from: https://bmjopen.bmj.com/content/bmjopen/9/12/e031763/DC2/embed/inline-supplementary-material-2.pdf?download=true doi: 10.1136/bmjopen-2019-031763 31852700 PMC6937101

[18] Aelbrecht K, Rimondini M, Bensing J, Moretti F, Willems S, Mazzi M, et al. Quality of doctor–patient communication through the eyes of the patient: variation according to the patient’s educational level. Advances in Health Sciences Education. 2014 Nov 27;20(4):873–84. doi: 10.1007/s10459-014-9569-6 25428194

[19] Aseltine RH, Sabina A, Barclay G, Graham G. Variation in patient–provider communication by patient’s race and ethnicity, provider type, and continuity in and site of care: An analysis of data from the Connecticut Health Care Survey. SAGE Open Medicine. 2016 Jan;4:205031211562516. 10.1177/2050312115625162 PMC4724761 26835017

[20] Hilton B, Tempest-Mitchell J, Davies B, Kotter M. Route to diagnosis of degenerative cervical myelopathy in a UK healthcare system: a retrospective cohort study. BMJ Open. 2019 May;9(5):e027000. doi: 10.1136/bmjopen-2018-027000 31061045 PMC6501948

[21] Moghaddamjou A, Wilson JRF, Martin AR, Gebhard H, Fehlings MG. Multidisciplinary approach to degenerative cervical myelopathy. Expert Review of Neurotherapeutics. 2020 Aug 4;20(10):1037–46. doi: 10.1080/14737175.2020.1798231 32683993

[22] Tempest-Mitchell J, Hilton B, Davies BM, Nouri A, Hutchinson PJ, Scoffings DJ, et al. A comparison of radiological descriptions of spinal cord compression with quantitative measures, and their role in non-specialist clinical management. Sherman JH, editor. PLOS ONE. 2019 Jul 22;14(7):e0219380. doi: 10.1371/journal.pone.0219380 31329621 PMC6645712

[23] Hilton B, Tempest-Mitchell J, Davies BM, Francis J, Mannion RJ, Trivedi R, et al. Cord compression defined by MRI is the driving factor behind the decision to operate in Degenerative Cervical Myelopathy despite poor correlation with disease severity. Farag E, editor. PLOS ONE. 2019 Dec 26;14(12):e0226020. doi: 10.1371/journal.pone.0226020 31877151 PMC6932812

[24] Klerings I, Weinhandl AS, Thaler KJ. Information overload in healthcare: too much of a good thing? Zeitschrift fur Evidenz, Fortbildung und Qualitat im Gesundheitswesen [Internet]. 2015;109(4–5):285–90. Available from: https://www.ncbi.nlm.nih.gov/pubmed/26354128 doi: 10.1016/j.zefq.2015.06.005 26354128

[25] Main BG, McNair AGK, Huxtable R, Donovan JL, Thomas SJ, Kinnersley P, et al. Core information sets for informed consent to surgical interventions: baseline information of importance to patients and clinicians. BMC Medical Ethics. 2017 Apr 26;18(1). doi: 10.1186/s12910-017-0188-7 28446164 PMC5406972

[26] Ha JF, Longnecker N. Doctor-patient communication: a review. The Ochsner journal [Internet]. 2010;10(1):38–43. Available from: https://www.ncbi.nlm.nih.gov/pmc/articles/PMC3096184/ 21603354 PMC3096184

[27] Fagan P, Harden B, Iongh A, Wright C. Person-Centred Approaches: Empowering people in their lives and communities to enable an upgrade in prevention, wellbeing, health, care and support. NHS Health Education England, Skills for Health, and Skills for Care. https://www.skillsforhealth.org.uk/images/pdf/Person-Centred-Approaches-Framework.pdf?s=form. 2017.

[28] General Medical Council. Decision making and consent [Internet]. www.gmc-uk.org. 2020. Available from: https://www.gmc-uk.org/-/media/documents/updated-decision-making-and-consent-guidance_pdf-84160128.pdf

[29] Personalised Care Institute. Curriculum August 2020. [Internet]. https://www.personalisedcareinstitute.org.uk/. 2020 Aug. Available from: https://www.personalisedcareinstitute.org.uk/pluginfile.php/133/mod_page/content/22/PCI_Curriculum.pdf

[30] Davies B, Sadler I, Widdop S. Poverty & Myelopathy [Internet]. Myelopathy Matters; 2021. Available from: https://podcasts.apple.com/gb/podcast/series-2-ep-1-poverty-myelopathy/id1493647316?i=1000508431282

[31] Atlas A, Milanese S, Grimmer K, Barras S, Stephens JH. Sources of information used by patients prior to elective surgery: a scoping review. BMJ Open. 2019 Aug;9(8):e023080. doi: 10.1136/bmjopen-2018-023080 31383690 PMC6687002

[32] Sustersic M, Gauchet A, Foote A, Bosson J-L. How best to use and evaluate Patient Information Leaflets given during a consultation: a systematic review of literature reviews. Health Expectations [Internet]. 2016 Sep 26;20(4):531–42. Available from: https://onlinelibrary.wiley.com/doi/full/10.1111/hex.12487 27669682 10.1111/hex.12487 PMC5512995

[33] Protheroe J, Estacio EV, Saidy-Khan S. Patient information materials in general practices and promotion of health literacy: an observational study of their effectiveness. British Journal of General Practice. 2015 Mar;65(632):e192–7. doi: 10.3399/bjgp15X684013 25733441 PMC4337308

[34] Tonsaker T, Bartlett G, Trpkov C. Health information on the Internet. Canadian Family Physician [Internet]. 2014 May 1 [cited 2021 Apr 18];60(5):407–8. Available from: http://www.ncbi.nlm.nih.gov/pmc/articles/pmc4020634/ 24828994 PMC4020634

[35] Tan SS-L, Goonawardene N. Internet Health Information Seeking and the Patient-Physician Relationship: A Systematic Review. Journal of Medical Internet Research. 2017 Jan 19;19(1):e9. doi: 10.2196/jmir.5729 28104579 PMC5290294

[36] Madathil KC, Rivera-Rodriguez AJ, Greenstein JS, Gramopadhye AK. Healthcare information on YouTube: A systematic review. Health informatics journal [Internet]. 2015;21(3):173–94. Available from: https://www.ncbi.nlm.nih.gov/pubmed/24670899 doi: 10.1177/1460458213512220 24670899

[37] Khan M, Mowforth O, Kuhn I, Kotter M, Davies B. Development of a validated search filter for Ovid Embase for degenerative cervical myelopathy. Wiley [Internet]. 2021 Aug 19; Available from: https://pubmed.ncbi.nlm.nih.gov/34409722/ doi: 10.1111/hir.12373 34409722

[38] Davies BM, Goh S, Yi K, Kuhn I, Kotter MRN. Development and validation of a MEDLINE search filter/hedge for degenerative cervical myelopathy. BMC Medical Research Methodology. 2018 Jul 6;18(1). doi: 10.1186/s12874-018-0529-3 29976134 PMC6034255

[39] Search engine market share worldwide [Internet]. Statista. Available from: https://www.statista.com/statistics/216573/worldwide-market-share-of-search-engines/#:~:text=Google%20has%20dominated%20the%20search

[40] Davies BM, Khan DZ, Mowforth OD, McNair AGK, Gronlund T, Kolias AG, et al. RE-CODE DCM (REsearch Objectives and Common Data Elements for Degenerative Cervical Myelopathy): A Consensus Process to Improve Research Efficiency in DCM, Through Establishment of a Standardized Dataset for Clinical Research and the Definition of the Research Priorities. Global Spine Journal. 2019 May;9(1_suppl):65S76S. doi: 10.1177/2192568219832855 31157148 PMC6512197

[41] Alexa—Top Sites by Category: Top/Health/Education. [Internet]. www.alexa.com. Available from: https://www.alexa.com/topsites/category/Top/Health/Education

[42] Hutton M. Spinal Services GIRFT Programme National Specialty Report. Appendix C: Specialised Providers of Complex Spinal Surgery (2017/18). 2019.

[43] CSM Information for Patients [Internet]. MYELOPATHY.ORG. [cited 2021 Jan 17]. Available from: https://myelopathy.org/csm-information-for-patients

[44] Nouri A, Tetreault L, Singh A, Karadimas SK, Fehlings MG. Degenerative Cervical Myelopathy: Epidemiology, Genetics, and Pathogenesis. Spine. 2015 Jun;40(12):E675–93. doi: 10.1097/BRS.0000000000000913 25839387

[45] Karadimas SK, Erwin WM, Ely CG, Dettori JR, Fehlings MG. Pathophysiology and Natural History of Cervical Spondylotic Myelopathy. Spine. 2013 Oct;38:S21–36. doi: 10.1097/BRS.0b013e3182a7f2c3 23963004

[46] Martin AR, De Leener B, Cohen-Adad J, Kalsi-Ryan S, Cadotte DW, Wilson JR, et al. Monitoring for myelopathic progression with multiparametric quantitative MRI. Toft M, editor. PLOS ONE. 2018 Apr 17;13(4):e0195733. doi: 10.1371/journal.pone.0195733 29664964 PMC5903654

[47] Yonenobu K, Abumi K, Nagata K, Taketomi E, Ueyama K. Interobserver and Intraobserver Reliability of the Japanese Orthopaedic Association Scoring System for Evaluation of Cervical Compression Myelopathy. Spine. 2001 Sep;26(17):1890–4. doi: 10.1097/00007632-200109010-00014 11568701

[48] Bartels RHMA, Verbeek ALM, Benzel EC, Fehlings MG, Guiot BH. Validation of a translated version of the modified Japanese orthopaedic association score to assess outcomes in cervical spondylotic myelopathy: an approach to globalize outcomes assessment tools. Neurosurgery [Internet]. 2010 May 1 [cited 2022 Mar 24];66(5):1013–6. Available from: https://pubmed.ncbi.nlm.nih.gov/20404709/ doi: 10.1227/01.NEU.0000368391.79314.6F 20404709

[49] Tetreault LA, Karpova A, Fehlings MG. Predictors of outcome in patients with degenerative cervical spondylotic myelopathy undergoing surgical treatment: results of a systematic review. European Spine Journal. 2013 Feb 6;24(S2):236–51. doi: 10.1007/s00586-013-2658-z 23386279

[50] Kato S, Ganau M, Fehlings MG. Surgical decision-making in degenerative cervical myelopathy–Anterior versus posterior approach. Journal of Clinical Neuroscience. 2018 Dec;58:7–12. doi: 10.1016/j.jocn.2018.08.046 30279123

[51] Ganau M, Holly LT, Mizuno J, Fehlings MG. Future Directions and New Technologies for the Management of Degenerative Cervical Myelopathy. Neurosurgery Clinics of North America. 2018 Jan;29(1):185–93. doi: 10.1016/j.nec.2017.09.006 29173432

[52] Cutilli CC. Seeking health information: what sources do your patients use? Orthopedic nursing [Internet]. 2010 [cited 2019 May 12];29(3):214–9. Available from: https://www.ncbi.nlm.nih.gov/pubmed/20505493 doi: 10.1097/NOR.0b013e3181db5471 20505493

[53] Mowforth OD, Davies BM, Goh S, O’Neill CP, Kotter MRN. Research Inefficiency in Degenerative Cervical Myelopathy: Findings of a Systematic Review on Research Activity Over the Past 20 Years. Global Spine Journal. 2019 Jun 12;10(4):476–85. doi: 10.1177/2192568219847439 32435569 PMC7222686

[54] Zileli M. Recommendations of WFNS Spine Committee. Neurospine. 2019 Sep 30;16(3):383–5. doi: 10.14245/ns.19int003 31607070 PMC6790736

[55] Boerger TF, Davies BM, Sadler I, Sarewitz E, Kotter MRN. Patient, sufferer, victim, casualty or person with cervical myelopathy: let us decide our identifier. Integrated Healthcare Journal. 2020 Jun;2(1):e000023. 10.1136/ihj-2019-000023 PMC10327448 37441311

[56] Davies B, Mowforth O, Sadler I, Aarabi B, Kwon B, Kurpad S, et al. Recovery priorities in degenerative cervical myelopathy: a cross-sectional survey of an international, online community of patients. BMJ Open. 2019 Oct;9(10):e031486. doi: 10.1136/bmjopen-2019-031486 31601597 PMC6797315

[57] Davies BM, McHugh M, Elgheriani A, Kolias AG, Tetreault LA, Hutchinson PJA, et al. Reported Outcome Measures in Degenerative Cervical Myelopathy: A Systematic Review. Ahmad F, editor. PLOS ONE. 2016 Aug 2;11(8):e0157263. doi: 10.1371/journal.pone.0157263 27482710 PMC4970758

[58] Davies BM, McHugh M, Elgheriani A, Kolias AG, Tetreault L, Hutchinson PJA, et al. The reporting of study and population characteristics in degenerative cervical myelopathy: A systematic review. Grasso G, editor. PLOS ONE. 2017 Mar 1;12(3):e0172564. doi: 10.1371/journal.pone.0172564 28249017 PMC5332071

[59] Boogaarts HD, Bartels RHMA. Prevalence of cervical spondylotic myelopathy. European Spine Journal. 2013 Apr 25;24(S2):139–41. doi: 10.1007/s00586-013-2781-x 23616201

[60] Butler MB, Mowforth OD, Badran A, Starkey M, Boerger T, Sadler I, et al. Provision and Perception of Physiotherapy in the Nonoperative Management of Degenerative Cervical Myelopathy (DCM): A Cross-Sectional Questionnaire of People Living With DCM. Global Spine Journal. 2020 Oct 1;219256822096135. doi: 10.1177/2192568220961357 33000656 PMC9109573

[61] Oh T, Lafage R, Lafage V, Protopsaltis T, Challier V, Shaffrey C, et al. Comparing Quality of Life in Cervical Spondylotic Myelopathy with Other Chronic Debilitating Diseases Using the Short Form Survey 36-Health Survey. World Neurosurgery. 2017 Oct;106:699–706. doi: 10.1016/j.wneu.2016.12.124 28065875

[62] Cost of myelopathy to society. www.myelopathy.org.uk. Unpublished.

[63] Main BG, McNair AGK, Haworth S, Rooshenas L, Hughes CW, Tierney P, et al. Core information set for informed consent to surgery for oral or oropharyngeal cancer: A mixed-methods study. Clinical Otolaryngology. 2017 Dec 21;43(2):624–31. doi: 10.1111/coa.13037 29178168

[64] Blazeby JM, Macefield R, Blencowe NS, Jacobs M, McNair AGK, Sprangers M, et al. Core information set for oesophageal cancer surgery. British Journal of Surgery. 2015 May 18;102(8):936–43. doi: 10.1002/bjs.9840 25980524

[65] McNair AGK, Whistance RN, Main B, Forsythe R, Macefield R, Rees J, et al. Development of a core information set for colorectal cancer surgery: a consensus study. BMJ Open. 2019 Nov;9(11):e028623. doi: 10.1136/bmjopen-2018-028623 31727644 PMC6886994

[66] Davies BM, Munro C, Khan DZ, Fitzpatrick SM, Hilton B, Mowforth OD, et al. Outcomes of Degenerative Cervical Myelopathy From The Perspective of Persons Living With the Condition: Findings of a Semistructured Interview Process With Partnered Internet Survey. Global Spine Journal. 2020 Nov 18;219256822095381. doi: 10.1177/2192568220953811 33203262 PMC9121154

[67] Khan DZ, Fitzpatrick SM, Hilton B, McNair AG, Sarewitz E, Davies BM, et al. Prevailing Outcome Themes Reported by People With Degenerative Cervical Myelopathy: Focus Group Study. JMIR Formative Research. 2021 Feb 3;5(2):e18732. doi: 10.2196/18732 33533719 PMC7889422

[68] Clarke MA, Moore JL, Steege LM, Koopman RJ, Belden JL, Canfield SM, et al. Health information needs, sources, and barriers of primary care patients to achieve patient-centered care: A literature review. Health Informatics Journal [Internet]. 2016 Jul 26;22(4):992–1016. Available from: https://nebraska.pure.elsevier.com/en/publications/health-information-needs-sources-and-barriers-of-primary-care-pat doi: 10.1177/1460458215602939 26377952

[69] Swoboda CM, Van Hulle JM, McAlearney AS, Huerta TR. Odds of talking to healthcare providers as the initial source of healthcare information: updated cross-sectional results from the Health Information National Trends Survey (HINTS). BMC Family Practice. 2018 Aug 29;19(1). doi: 10.1186/s12875-018-0805-7 30157770 PMC6116497

[70] Kenny T. A PIL for every ill? Patient information leaflets (PILs): a review of past, present and future use. Family Practice. 1998 Oct 1;15(5):471–9. doi: 10.1093/fampra/15.5.471 9848435

[71] Grodzinski B, Bestwick H, Bhatti F, Durham R, Khan M, Partha Sarathi CI, et al. Research activity amongst DCM research priorities. Acta Neurochirurgica. 2021 Feb 24; doi: 10.1007/s00701-021-04767-6 33625603 PMC8116279

[72] Theodore N. Degenerative Cervical Spondylosis. Ropper AH, editor. New England Journal of Medicine. 2020 Jul 9;383(2):159–68. doi: 10.1056/NEJMra2003558 32640134

[73] Waqar M, Wilcock J, Garner J, Davies B, Kotter M. Quantitative analysis of medical students’ and physicians’ knowledge of degenerative cervical myelopathy. BMJ Open. 2020 Jan;10(1):e028455. doi: 10.1136/bmjopen-2018-028455 31932384 PMC7044983

[74] Davies BM, Munro CF, Kotter MR. A Novel Insight Into the Challenges of Diagnosing Degenerative Cervical Myelopathy Using Web-Based Symptom Checkers. Journal of Medical Internet Research. 2019 Jan 11;21(1):e10868. doi: 10.2196/10868 30300137 PMC6330198

[75] Colledge A, Car J, Donnelly A, Majeed A. Health information for patients: time to look beyond patient information leaflets. Journal of the Royal Society of Medicine [Internet]. 2008 Sep;101(9):447–53. Available from: https://www.ncbi.nlm.nih.gov/pmc/articles/PMC2587380/ doi: 10.1258/jrsm.2008.080149 18779246 PMC2587380

