# Supplementary material for: A scoping review of information provided within degenerative cervical myelopathy education resources: Towards enhancing shared decision making
Source: PLoS One. 2022 May 19;17(5):e0268220. doi: 10.1371/journal.pone.0268220 (PMC9119544; doi:10.1371/journal.pone.0268220)
Supplement: S1 Appendix — (DOCX) [file pone.0268220.s001.docx]

**S1 Appendix. Search strategy identifying educational resources in scientific literature, videos, organisations, health education websites and hospital patient information leaflets**

**1. Scientific literature**

**a. Systematic Reviews**

**Table 1 – Search strategy for identifying systematic reviews with educational DCM content from scientific literature databases**

| Database | Search Term | Filters selected | Rationale for search |
| --- | --- | --- | --- |
| EMBASE | EMBASE DCM filter | Human AND EBM (Evidence Based Medicine) – Systematic Review AND Years Published – Last 20 years | Clinical query raised by a systematic review, is likely to represent a key clinical question and therefore information that should be relayed in a core information set |
| MEDLINE | MEDLINE DCM filter | Human AND Publication Type – Systematic Review AND Year Published – Last 20 years |  |
| PROSPERO | Myelopathy | N/A | Indication of any ongoing / unpublished systematic review in myelopathy |

**b. Narrative Reviews**

**Table 2 – Search strategy for identifying narrative reviews with educational DCM content from scientific literature databases**

| Database | Search Term | Filters selected | Rationale for search |
| --- | --- | --- | --- |
| EMBASE | EMBASE DCM Filter | Human AND Publication Type – Review AND Year Published – Last 3 years | Narrative reviews from last 3 years are professional educational articles and will represent the core evidence-based information being disseminated for professionals |
| MEDLINE | MEDLINE DCM Filter | Human AND Publication Type – Review AND Year Published – Last 3 years |  |

**2. Videos**

**Table 3 – Search strategy for identifying videos with educational DCM content from online database of videos**

| Database | Search | Rationale for search |
| --- | --- | --- |
| Google | Cervical Myelopathy | Search of Google videos provides good breadth of videos on DCM |

**3. Organisations**

**Table 4a – Search strategy for identifying a list of organisation websites with educational DCM content from database of organisations**

| Database | Search | Rationale for search |
| --- | --- | --- |
| ENVIROSCAN | Cervical myelopathy | Global list of organisations, including charities, hospitals and universities and professional bodies. This includes organisations already doing work related to DCM and those that have the potential to do work related to DCM |

**Table 4b – Hierarchical search strategy to identify educational content on DCM from organisation websites. Method 1 was employed first. Method 2 was employed if no information on DCM was found using method 1. Method 3 was used employed if no information was found using methods 1 and 2. If no information found using all 3 methods, the resources was excluded.**

| **Method** | **Tool** | **Search term** | **Additional information** |
| --- | --- | --- | --- |
| 1 | From navigation menu select webpage for information resources | Cervical myelopathy | N/A |
| 2 | Searchbar on website | Cervical myelopathy | Search performed once for a given website |
| 3 | Find in page function (Ctrl + F) | Cervical myelopathy | Search repeated for each webpage page on a given website |

**4. Health Education Websites**

**Table 5a – Search strategy for generating a list of health education websites with educational DCM content from online database of websites**

| Database | Search | Rationale for search |
| --- | --- | --- |
| Alexa Top Site, Amazon Web Service | Cervical myelopathy | List of the most popular websites under the category health, subcategory education, likely to contain educational resources on DCM |

**Table 5b – Hierarchical search strategy to identify educational content on DCM from health education websites. Method 1 was employed first. Method 2 was employed if no information on DCM was found using method 1. Method 3 was used employed if no information was found using methods 1 and 2. If no information found using all 3 methods, the resources was excluded.**

| **Method** | **Tool** | **Search term** | **Additional information** |
| --- | --- | --- | --- |
| 1 | From navigation menu select educational resources webpage AND/OR patient webpage then resources AND/OR professional webpage then resources | Cervical myelopathy | N/A |
| 2 | Searchbar on website | Cervical myelopathy | Search performed once for a given website |
| 3 | Find in page function (Ctrl + F) | Cervical myelopathy | Search repeated for each webpage page on a given website |

**5. Hospital Patient Information Leaflets**

**Table 6a – Search strategy for identifying hospital patient information leaflets with educational DCM content from online database of hospitals**

| Database | Search | Rationale for search |
| --- | --- | --- |
| Hospital Providers of Complex Spinal Surgery | Cervical myelopathy | Spinal Services GIRFT Programme National Specialty Report, Appendix C, provides a list of hospitals that provide spinal services and would be involved in management of patients with DCM in the UK |

**Table 6b – Hierarchical search strategy to identify educational content on DCM from patient information leaflets from hospital websites. Method 1 was employed first. Method 2 was employed if no information on DCM was found using method 1. Method 3 was used employed if no information was found using methods 1 and 2. If no information found using all 3 methods, the resources was excluded.**

| **Method** | **Tool** | **Search term** | **Additional information** |
| --- | --- | --- | --- |
| 1 | From hospital website navigation menu select webpage for patients then patient information leaflets OR patient information leaflets webpage | Cervical myelopathy | N/A |
| 2 | Searchbar on website | Cervical myelopathy | Search performed once for a given website |
| 3 | Find in page function (Ctrl + F) | Cervical myelopathy | Search repeated for each webpage page on a given website |
