# Supplementary material for: A scoping review of information provided within degenerative cervical myelopathy education resources: Towards enhancing shared decision making
Source: PLoS One. 2022 May 19;17(5):e0268220. doi: 10.1371/journal.pone.0268220 (PMC9119544; doi:10.1371/journal.pone.0268220)
Supplement: S2 Appendix — (DOCX) [file pone.0268220.s002.docx]

**S2 Appendix. Inclusion and exclusion criteria for educational resources in scientific literature, videos, organisations, health education websites and hospital patient information leaflets**

**1. Scientific Literature**

**a. Systematic review**

**Table 1 - Specific inclusion and exclusion for systematic reviews for screening resources to identify those with educational DCM content**

| Inclusion Criteria | Exclusion criteria |
| --- | --- |
| Written in English  Degenerative Cervical Myelopathy  Cervical Spondylotic Myelopathy +/- OPLL | Heterogenous populations (ie not exclusively DCM or CSM+/- OPLL)  Cervical radiculopathy  Cervical myelopathy of other aetiology  Cervical degenerative disc disease without myelopathy  OPLL without myelopathy  Cervical compressive myelopathy? |

**b. Narrative Review**

**Table 2 - Specific inclusion and exclusion for narrative reviews for screening resources to identify those with educational DCM content**

| Inclusion Criteria | Exclusion Criteria |
| --- | --- |
| Full text article written in English  Educational tool overview for professionals  Degenerative cervical myelopathy  Cervical spondylotic myelopathy +/- OPLL | Heterogenous populations (ie not exclusively DCM or CSM+/- OPLL)  Cervical radiculopathy  Systematic review |

**2. Videos**

**Table 3 - Specific inclusion and exclusion for videos for screening resources to identify those with educational DCM content**

| Inclusion Criteria | Exclusion criteria |
| --- | --- |
| Educational Videos for Patients or Professionals  Video in English | Advertisements / Promotional Video  Operative Technique Videos  Different Pathology  *Cervical Stenosis*  *Cervical Radiculopathy*  Non-Evidence Based Treatment [Not listed in international guidelines (E.g. Yoga, Herbal Remedies….) ]  Examination Findings (signs/reflexes) |

**3. Organisations**

**Table 4 - Specific inclusion and exclusion for organisations for screening resources to identify those with educational DCM content**

| Inclusion Criteria | Exclusion criteria |
| --- | --- |
| Educational Information for Patients or Professionals  Webpage written in English | Reported on heterogenous populations (not exclusively DCM or CSM ± OPLL)  Different Pathology  *Cervical Stenosis*  *Cervical Radiculopathy*  Non-Evidence Based Treatment [Not listed in international guidelines (E.g. Yoga, Herbal Remedies….) ] |

**4. Health Education Websites**

**Table 5 - Specific inclusion and exclusion for health education websites for screening resources to identify those with educational DCM content**

| Inclusion Criteria | Exclusion criteria |
| --- | --- |
| Educational Information for Patients or Professionals  Webpage written in English | Reported on heterogenous populations (not exclusively DCM or CSM ± OPLL)  Different Pathology  *Cervical Stenosis*  *Cervical Radiculopathy*  Non-Evidence Based Treatment [Not listed in international guidelines (E.g. Yoga, Herbal Remedies….) ] |

**5. Hospital Patient Information Leaflets**

**Table 6 - Specific inclusion and exclusion for hospital patient information leaflets for screening resources to identify those with educational DCM content**

| Inclusion Criteria | Exclusion criteria |
| --- | --- |
| Dedicated DCM Patient information leaflet | Cervical stenosis  Cervical radiculopathy  Explanation of surgery without any details on condition  Consent forms for surgery |
