# Supplementary material for: A scoping review of information provided within degenerative cervical myelopathy education resources: Towards enhancing shared decision making
Source: PLoS One. 2022 May 19;17(5):e0268220. doi: 10.1371/journal.pone.0268220 (PMC9119544; doi:10.1371/journal.pone.0268220)
Supplement: S3 Appendix — (DOCX) [file pone.0268220.s003.docx]

**S3 Appendix. DCM search filter for scientific literature databases: EMBASE and MEDLINE**

**Table 1 – EMBASE search filter**

| **#** | **[EMBASE]** |
| --- | --- |
| **1** | exp *cervical vertebra/ or exp *cervical spinal cord/ or cervical.ti,ab. or exp *cervical spine/ or (phrenic nucleus or accessory nucleus).ti,ab. or exp *Japanese Orthopaedic Association score/ or Japanese Orthop?edic Association.ti,ab. or ("Japanese Orthop?edic Association" adj2 scor*).ti,ab. or (joa adj2 scor*).ti,ab. |
| **2** | myelopath*.ti,ab. or exp cervical myelopathy/ or exp *cervical spondylotic myelopathy/ or spondylotic cervical myelopathy.mp. or exp *spinal cord disease/ or "cervical spinal cord injury".ti,ab. or exp *myelography/ or exp *myeloradiculopathy/ or myeloradiculopath*.ti,ab. or exp *cervical spondylosis/ or (spinal cord adj3 (diseas* or disorder*)).ti,ab. or spondylomyelopath*.ti,ab. or (Spinal Cord adj3 Compress*).ti,ab. or exp *spinal cord compression/ |
| **3** | 1 and 2 |
| **4** | "ossification of posterior longitudinal ligament".ti,ab. or exp *ligament calcinosis/ or (exp *posterior longitudinal ligament/ and (exp *ossification/ or ossifi*.ti,ab.)) |
| **5** | 3 or 4 |
| **6** | exp atlantooccipital joint/ or exp arteriovenous fistula/ or exp radiotherapy/ or exp cyanocobalamin/ or exp radiation injury repair/ or exp radiation injury/ or exp *radiation/ or exp re-irradiation/ or exp irradiation/ or exp craniospinal irradiation/ or exp whole body radiation/ or exp *motor neuron disease/ or exp *amyotrophic lateral sclerosis/ or neoplasm metastasis.mp. or exp metastasis/ or exp *neoplasm/ or exp malignant neoplasm/ or exp radiation induced neoplasm/ or exp myeloproliferative neoplasm/ or exp vertebra hemangioma/ or exp hemangioma/ or exp nervous system malformation/ or autoimmune diseases of the nervous system.mp. or autoimmune nervous system.mp. or (congenital, hereditary, and neonatal diseases and abnormalities).mp. or "congenital disorder".mp. or exp genetic disorder/ or "newborn disease".mp. or exp virus infection/ |
| **7** | 5 not 6 |

**Table 2 – MEDLINE search filter**

| **#** | **[MEDLINE]** |
| --- | --- |
| **1** | exp Cervical Vertebrae/ or exp Cervical Cord/ or cervical.mp. or (phrenic nucleus or accessory nucleus).mp. or (("Japanese Orthop?edic Association" adj2 score*) or (joa adj2 score*)).mp. |
| **2** | myelopath*.mp. or exp Spinal Cord Diseases/ or (spinal cord adj3 (diseas* or disorder*)).mp. or myeloradiculopath*.mp. or spondylomyelopath*.mp. or spondylomyeloradiculopath*.mp. or (Spinal Cord adj3 Compress*).mp. or exp Spinal Cord Compression/ |
| **3** | 1 and 2 |
| **4** | exp "Ossification of Posterior Longitudinal Ligament"/ |
| **5** | 3 or 4 |
| **6** | exp Atlanto-Occipital Joint/ or exp Arteriovenous Fistula/ or exp Radiotherapy/ or exp Vitamin B 12/ or exp Radiation/ or exp Radiation Injuries/ or exp Re-Irradiation/ or exp Craniospinal Irradiation/ or exp Whole-Body Irradiation/ or exp Motor Neuron Disease/ or exp Amyotrophic Lateral Sclerosis/ or exp Neoplasm Metastasis/ or exp Hemangioma/ or exp neoplasm/ or exp metastasis/ or exp Nervous System Malformations/ or exp "autoimmune diseases of the nervous system"/ or exp "congenital, hereditary, and neonatal diseases and abnormalities"/ ors exp virus diseases/ |
| **7** | 5 not 6 |
