# Supplementary material for: A scoping review of information provided within degenerative cervical myelopathy education resources: Towards enhancing shared decision making
Source: PLoS One. 2022 May 19;17(5):e0268220. doi: 10.1371/journal.pone.0268220 (PMC9119544; doi:10.1371/journal.pone.0268220)
Supplement: S4 Appendix — (DOCX) [file pone.0268220.s004.docx]

**S4 Appendix. List of UK hospitals offering complex spinal surgery services – taken from Spinal Services GIRFT Programme National Specialty Report - Appendix C**

Alder Hey Children’s NHS Foundation Trust

Barking, Havering and Redbridge University Hospitals NHS Trust

Barts Health NHS Trust

Brighton and Susses University Hospitals NHS Trust

Buckinghamshire Healthcare NHS Trust

Cambridge University Hospitals NHS Foundation Trust

Central Manchester University Hospitals NHS Foundation Trust

Derby Teaching Hospitals NHS Foundation Trust

Great Ormond Street Hospital for Children NHS Foundation Trust

Guy’s and St Thomas’ NHS Foundation Trust

Hull and East Yorkshire Hospitals NHS Trust

Ipswich Hospital NHS Trust

King’s College Hospitals NHS Foundation Trust

Lancashire Teaching Hospitals NHS Foundation Trust

Leeds Teaching Hospitals NHS Trust

Norfolk and Norwich University Hospitals NHS Foundation Trust

North Bristol NHS Trust

North Tees and Hartlepool NHS Foundation Trust

Nottingham University Hospitals NHS Trust

Oxford University Hospitals NHS Foundation Trust

Plymouth Hospitals NHS Trust

Royal Devon and Exeter NHS Foundation Trust

Royal National Orthopaedic Hospital NHS Trust

Salford Royal NHS Foundation Trust

Sheffield Children’s NHS Foundation Trust

Sheffield Teaching Hospitals NHS Foundation Trust

South Tees Hospitals NHS Foundation Trust

St George’s University Hospitals NHS Foundation Trust

Taunton and Somerset NHS Foundation Trust

The Newcastle Upon Tyne Hospitals NHS Foundation Trust

The Robert Jones and Agnes Hunt Orthopaedic Hospital NHs Foundation Trust The Royal Orthopaedic Hospital NHS Foundation Trust

The Walton Centre NHS Foundation Trust

University College London Hospitals NHS Foundation Trust

University Hospitals Southampton NHS Foundation Trust

University Hospitals Birmingham NHS Foundation Trust

University Hospitals Bristol NHS Foundation Trust

University Hospitals Coventry and Warwickshire NHS Trust

University Hospitals of Leicester NHS Trust

University Hospitals of North Midlands NHS Trust
